# Cyberbullying detection approaches for Arabic texts: a systematic literature review

**DOI:** 10.3389/frai.2025.1666349

**Published:** 2025-10-16

**Authors:** Hooayda Allwaibed, Mohammed Anbar, Selvakumar Manickam, Annisa Bintang

**Affiliations:** ^1^Cybersecurity Research Centre (CYRES), Universiti Sains Malaysia (USM), Penang, Malaysia; ^2^Department of Computer Science, Applied College, Northern Border University, Arar, Saudi Arabia; ^3^Universitas Indonesia Fakultas Ilmu Komputer, Depok, Indonesia

**Keywords:** cyberbullying detection, Arabic language, systematic literature review, machine learning, deep learning, support vector machines, convolutional neural networks, natural language processing

## Abstract

This study presents a comprehensive review of current methodologies, trends, and challenges in cyberbullying detection within Arabic-language contexts, with a focus on the unique linguistic and cultural factors associated with Arabic. This study reviews 35 peer-reviewed articles about the identification of cyberbullying in Arabic text. Reported accuracies across datasets and platforms range from approximately 73 to 96%, with precision frequently surpassing recall, suggesting that systems are more adept at identifying blatant bullying than at encompassing all pertinent instances. Methodologically, conventional machine learning utilizing Arabic-specific characteristics remains effective on smaller datasets, however deep neural architectures—especially CNN/BiLSTM—and transformer models like AraBERT yield superior outcomes when dialectal heterogeneity and orthographic noise are mitigated. Evaluation methodologies differ; research using a neutral class frequently indicates exaggerated accuracy, underscoring the necessity to emphasize macro-averaged F1 and per-class metrics. The evidence underscores deficiencies in dialectal representativeness, the uniformity of bullying notions compared to general abuse, and the transparency of annotation processes. Ethical and deployment considerations—privacy preservation, dialectal bias, and real-time robustness—are becoming increasingly significant. We integrate trends (models and features), standards (labeling and metrics), and future work directions, encompassing dialect-robust pretraining, cross-dataset evaluation, context-aware modeling, and human-in-the-loop frameworks. The review offers a comprehensive basis for researchers and practitioners pursuing culturally and linguistically tailored approaches to Arabic cyberbullying detection.

## 1 Introduction

The extensive utilization of digital communication channels has resulted in a concerning rise in cyberbullying, a type of online harassment impacting persons of many age groups and demographics. This study evaluated the relevant research published from 2014 to 2024, to assess and contrast the efficacy of conventional machine learning methods, deep learning frameworks, and sentiment-oriented strategies in the classification of cyberbullying, highlighting the significance of linguistic and dialectal intricacies in detection precision.

IT communication platforms such as WhatsApp, Facebook Messenger, Viber, WeChat, Line, Telegram, Imo, and Kakao Talk have increased in use throughout the last years, with some having over 1.5 billion users (Urrutia Zubikarai, 2020). Several sources contended that offensive content in social media and communication platforms has become extremely dangerous; for instance, issues relating to social media in public institutions, particularly during the election period, are related to offensive content and have become challenging for public institutions in light of how information should be controlled (Grégoire et al., 2015). Offensive content, generally in the form of foul language spouting racial hate, personal attacks, and sexual harassment, is prevalent. Hence, it is important to detect offensive use of language to maintain a healthy discussion and enhance the security of users through the suppression of such hateful acts and offences (Bertini et al., 2021; Niraula et al., 2021). Online content-generators have increased, allowing more users to experience the freedom to express themselves, covered with anonymity if they choose, which maximizes the chance for platform misuse and leads to an environment that promotes offensive language and even eventually violence (Sap et al., 2019). Also, social networking platforms display several types of offensive language like hate speech, aggressive content, cyberbullying, and toxic statements (Mirończuk and Protasiewicz, 2018). A possible way to curtail and control such a phenomenon is through the use of NLP techniques like text classification for the automatic detection of offensive language. More specifically, text classification is the process of labelling new text with pre-defined labels (Mirończuk and Protasiewicz, 2018).

## 2 Background of study

### 2.1 Cyberbullying

Cyberbullying has become a global concern with the rise of social media and online platforms, and research efforts are increasingly being devoted to detecting and mitigating it using Machine Learning (ML), Deep Learning (DL), and Natural Language Processing (NLP) approaches. While a significant amount of research has been conducted in languages like English, studies targeting cyberbullying in Arabic remain limited. This systematic literature review aims to explore existing research on cyberbullying detection in the Arabic language, with a focus on ML and DL techniques, and to identify future research directions based on the analysis of the reviewed studies.

### 2.2 Challenges in detecting in Arabic language

Identifying cyberbullying in the Arabic language poses difficulties, mostly due to the linguistic, cultural, and computational intricacies involved in processing Arabic content. A principal challenge is the significant range of Arabic dialects, which differ not only by area but also by socio-economic and cultural factors. Although Modern Standard Arabic (MSA) is extensively employed in formal discourse, social media exchanges primarily transpire in dialectal Arabic, which is characterized by the absence of standardized spelling, syntax, and vocabulary (Mubarak and Darwish, 2019; AbdelHamid et al., 2022). The lack of high-quality, labeled datasets that consider these changes intensifies the issue, resulting in diminished model performance in real-world Arabic cyberbullying detection tasks (Bashir and Bouguessa, 2021; Khairy et al., 2023). A fundamental problem is the morphological complexity and intricate syntax of Arabic, which markedly contrasts with Indo-European languages like English. Arabic lexicon demonstrates significant inflexion through affixation, root-based derivations, and contextual variants, complicating tokenization, stemming, and lemmatization (Alakrot et al., 2018; Haidar et al., 2019). The linguistic features create difficulty in text classification, as identical words may possess varying meanings based on diacritical marks, which are frequently absent in informal online communication. The scarcity of comprehensive pre-trained models tailored for Arabic dialects constrains the capacity of NLP algorithms to effectively identify harmful and abusive content (Alrashidi et al., 2023; Khezzar et al., 2023). Research indicates that sentiment analysis and lexicon-based methodologies can improve detection by identifying emotional indicators; however, their efficacy is limited by the necessity for manually curated lexicons specific to Arabic dialects (Farid and El-Tazi, 2020). An application of NLP that extracts structured information in the form of entities, entities’ relationship and attributes describing them from unstructured documents in an automatic method is Information Extraction (IE) (Cowie and Lehnert, 1996). Besides, IE systems have been found effective in handling information overload issues, enabling the discernment of the most significant information portion from a huge portion of information in a timely and easy manner. On the whole, detection of offensive language online is possible through the development of a model using ML, AI, DL and NLP methods. This paper investigates the following research questions:

## 3 Research questions

**Q1:** What are the current trends in cyberbullying detection for the Arabic language and which dialects do they cover?

**Q2:** How cyberbullying been detected in previous studies based on standards that represent its definition and characteristics?

**Q3:** What directions for future research in cyberbullying detection may be established based on the findings of this review?

## 4 Methodology

A systematic literature review was conducted to conduct a comprehensive analysis by focusing on existing studies from 2014 to 2024, evaluating trends and advancements in cyberbullying detection for Arabic texts. This methodology involves structured selection criteria to ensure that only relevant and high-quality sources are included. The Inclusion Criteria are as follows:

1. Studies published from 2014 to 2024
2. Articles in English
3. Research specific to Arabic text-based cyberbullying detection

The exclusion criteria were:

1. The research focused on social studies without technological elements
2. Studies in languages other than English and non-Arabic texts
3. Non-text-based detection methods (e.g., voice, image, video)
4. Conference papers and review articles

SLR protocol was applied to the study, the final selected studies were conducted, and theoretical and practical steps were taken while conducting the SLR.

## 5 Data sources and keywords

**Figure 1:**
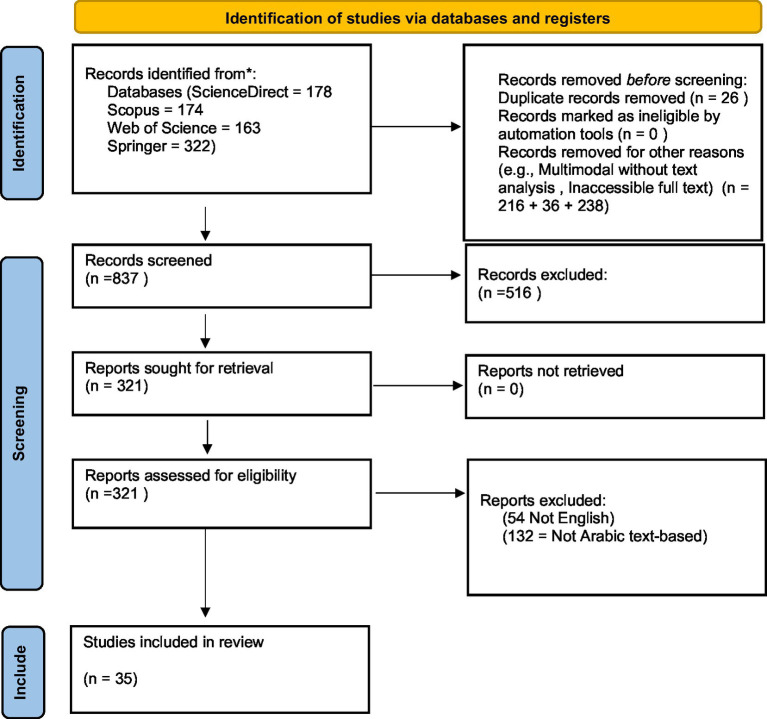
Systematic literature review process.

In the first step, four major research databases, ScienceDirect, Scopus, Web of Science, and Springer, were searched through queries, and as many papers as possible were collected. The search query is “detect” AND (“cyberbullying” OR “hate speech” OR “harassment” OR “offensive”) AND (“machine learning” OR “natural language processing” OR “deep learning”) AND “Arabic.” Based on initial exclusion criteria, papers were selected after carefully reading the abstracts of the papers in the second step. A final list of papers is prepared after reading the full articles and applying further exclusion criteria (35 papers). Figure 1 depicts the literature review process.

## 6 Results

This review synthesizes findings from numerous studies on cyberbullying detection within Arabic-language content, identifying the main trends, challenges, and methodologies, including ML, DL, and sentiment analysis. The majority of the studies concentrated on cyberbullying detection, offensive language detection, and hate speech identification. A significant portion of the research applied to social media platforms like Twitter and YouTube. The focus was largely on identifying cyberbullying in dialects such as Saudi Arabian Arabic, Egyptian Arabic, and the Levantine dialects. The most frequently used machine learning models included Naïve Bayes (NB), Support Vector Machine (SVM), and Random Forest (RF). For deep learning models, LSTM, CNN, and GRU were prominent. Ensemble techniques like stacking and boosting showed better performance compared to individual ML models. The datasets used in the reviewed studies varied widely in size, ranging from small manually annotated datasets to large datasets collected from social media. Many studies employed preprocessing techniques such as tokenization, stemming, lemmatization, and removal of hyperlinks or non-Arabic characters to clean the data before analysis. Preprocessing was critical in ensuring the effectiveness of the ML and DL models. Across the reviewed studies, model performance is generally strong, with traditional machine learning and deep learning approaches demonstrating reliable detection capabilities in Arabic cyberbullying contexts. Reported results indicate that precision commonly exceeds recall, suggesting that systems are better at correctly identifying bullying instances than capturing all relevant cases. This pattern appears in works employing classical classifiers as well as ensemble strategies, with examples including Egyptian-dialect tweet classification (Farid and El-Tazi, 2020), Naïve Bayes–based detection pipelines (Mouheb et al., 2019), offensive language identification in user-generated video comments (Alakrot et al., 2018), and ensemble machine learning frameworks that optimize the balance of precision and recall (Haidar et al., 2019). In terms of offensive language and cyberbullying detection, researchers identify various types of offensive language, each reflecting specific social, cultural, and regional sensitivities. Table 1 illustrates the types of offensive language used in Arabic studies on cyberbullying and offensive content

**Table 1:** Types of offensive language used in Arabic studies on cyberbullying and offensive content.

Type of Offensive Language	Description	Sources
Hate Speech	Language targeting specific groups based on religion, race, gender, or nationality. Includes:	Castaño-Pulgarín et al. (2021), Alsafari et al. (2020a, 2020b)
Insults and Personal Attacks	Abusive language aimed at degrading individuals, including name-calling, derogatory remarks, and personal insults about appearance, intelligence, or social status.	Alshalabi et al. (2024),
Profanity and Vulgar Language	Taboo words or phrases generally considered offensive, including swear words and obscenities that are often censored on public platforms.	Rosenbaum (2019)
Sexual Harassment	Inappropriate comments or sexually explicit content targeting individuals, often related to gender-based discrimination.	Abdelmonem (2015), Bouhlila (2019), Bertini et al. (2021), Niraula et al. (2021)
Bullying and Harassment	Repeated or persistent offensive behavior aimed at intimidating or humiliating someone, often through derogatory remarks about personal life or achievements.	Kanan et al. (2020)
Stereotyping and Discrimination	Generalizations that promote negative stereotypes about specific groups (e.g., based on age, nationality, profession). Includes implicit bias and discriminatory remarks.	Alsafari et al. (2020a, 2020b)
Mockery and Sarcasm	Humorous or sarcastic language used to belittle or degrade individuals or groups, often through irony or exaggeration, which can vary in offensiveness depending on context.	Abu Farha (2023).

### 6.1 Research question 1

The first research question was:

What are the current trends in cyberbullying detection for the Arabic language, and how do these trends account for various dialects?

The following themes were developed to answer the first research question 1:

#### 6.1.1 Machine learning (ML) and deep learning (DL) approaches

Several studies have utilized ML and DL algorithms to detect cyberbullying, with Support Vector Machine (SVM) and Naïve Bayes (NB) being frequently applied (e.g., Haidar et al., 2017; Alakrot et al., 2018). More recently, DL methods such as Convolutional Neural Networks (CNNs) and Recurrent Neural Networks (RNNs) have demonstrated improved performance due to their ability to capture context and semantic meanings in text (e.g., Haidar et al., 2018; Mouheb et al., 2019; Mohaouchane et al., 2019). Ensemble learning, where multiple models are combined to improve prediction accuracy, has shown promise in boosting performance. For instance, stacking, boosting, and bagging techniques have demonstrated better performance in detecting Arabic cyberbullying content (e.g., Haidar et al., 2018; Khairy et al., 2023; Table 2).

**Table 2:** Summary of reviewed studies on Arabic hate/offensive/cyberbullying detection.

No	Study	Model(s)	Dataset and Platform	Dialect/Domain	Performance Metrics	Limitations
1	Haidar et al. (2017)	Naïve Bayes, SVM	Posts (Twitter, Facebook, Formspring)	Saudi Arabic	NB: Precision 90.85%; SVM: Precision 0.815 (yes class)	Imbalanced dataset; few bullying instances; precision misleading
2	Haidar et al. (2018)	Feed-forward Neural Network (DL)	Twitter dataset (binary labels)	General Arabic	Validation accuracy 91.17% (7 hidden layers)	Limited to binary labels; dataset size not large
3	Alakrot et al. (2018)	SVM	YouTube comments	General Arabic	Precision 90.05%	Small dataset; not specific to cyberbullying
4	AlHarbi et al. (2019)	Lexicon + Sentiment Analysis (PMI, Chi-square, Entropy)	Tweets	Twitter (Saudi Arabic)	PMI accuracy 81% vs. Chi-square 62.11%	Lexicon-based; potential bias; dataset context-limited
5	Mubarak and Darwish (2019)	ML classifiers	Arabic tweets	General Arabic	High classification accuracy	Focused only on offensive, not cyberbullying
6	Farid and El-Tazi (2020)	Lexicon-based Sentiment Analysis + Emojis	Tweets in Modern Standard + Egyptian Dialect	Egyptian Arabic	Accuracy >73% for bullying hashtags	Lexicon limited; reliance on emojis and history
7	Alsafari et al. (2020b)	LR, LSTM, Sluice, BERT, ELMo, SVM	Labeled tweets (Twitter)	Mixed Arabic dialects	SVM + ngrams: Acc. 85.16%; CNN + mBERT F1-macro 66.86%	Limited samples per class; subjectivity in annotation
8	Bashir and Bouguessa (2021)	LSTM, SVM, Naïve Bayes	Twitter dataset (cyberbullying keywords)	General Arabic	LSTM accuracy 72%	Keyword-based data collection; lower accuracy
9	Fati (2022)	Sentiment Analysis Framework	Twitter	General Arabic	Accuracy 81% (10-fold CV)	Limited validation; binary annotation
10	Al-Hassan and Al-Dossari (2022)	LSTM, CNN + LSTM, GRU, CNN + GRU	Labeled tweets	General Arabic	CNN + LSTM: Precision 72%, Recall 75%, F1 73%	Moderate dataset size; limited categories
11	Alsubait and Alfageh (2021)	Multinomial NB, Complement NB, Logistic Regression	YouTube comments	General Arabic	Avg. F1: TF-IDF 77.9% vs. CountVec 77.5%	Dataset modest; no deep learning comparison
12	Alhashmi and Darem (2022)	RF, NB, SVM, XGB, ANN, Stacked DL; Consensus-Based Ensemble	(Twitter, WhatsApp, Vine, Instagram, Packet; incl. Translated data)	Mixed Arabic + translated	Consensus ensemble improved accuracy by 1.3% over best classifier; RF strongest	Dataset partly translated; mixed domains; modest gain over baselines
13	Bouliche and Rezoug (2022)	Dynamic Graph Neural Network (DGNN)	Arabic comments (tweets)	General Arabic	Accuracy 74%	Model performance modest; needs refinement; small dataset
14	El-Alami et al. (2022)	BERT (multilingual, transfer learning)	Bilingual dataset (English + Arabic tweets)	General Arabic + English	High accuracy and F1; BERT outperformed other models	Ambiguous language still difficult; early-stage
15	AbdelHamid et al. (2022)	AraBERT, ArabicBERT, GigaBERT vs. RF, SVM	Syrian/Levantine tweets	Levantine dialect	GigaBERT: AUC 94.6%, Macro F1 0.81	Focused on Levantine; dataset scope limited
16	AlFarah et al. (2022)	SVM, RF, NB, LR, KNN	Twitter + YouTube, oversampled	General Arabic	NB highest AUC 89%; SVM and LR also strong	Class imbalance; dataset moderate in size
17	Anezi (2022)	Deep Recurrent Neural Network (DRNN)	Custom Arabic comments dataset	General Arabic	Binary Acc 99.73%; 3-class Acc 95.38%; 7-class Acc 84.14%	Dataset unique but limited disclosure; overfitting risk
18	Althobaiti (2022)	BERT + Sentiment + Emoji features vs. SVM, LR	Arabic tweets	General Arabic	BERT model highest F1 across all tasks	Single dataset; limited external validation
19	Ali and Kurdy (2022)	SVM, SGD, KNN, LR, AdaBoost, Bagging	Syrian Facebook comments + questionnaire	Syrian slang	SVM and SGD accuracy 77%; AdaBoost precision 94%	Imbalanced recall (47%); small dataset
20	Alduailaj and Belghith (2023)	SVM + FarasaNLTK vs. NB	Twitter + YouTube comments	General Arabic	SVM best accuracy 95.74% (TF-IDF n-gram)	Keyword-based collection; possible bias
21	Khairy et al. (2023)	Ensemble (Voting) vs. LR, SVC, KNN	New balanced dataset	General Arabic	Voting model highest Acc, F1, Recall, Precision; LR best single Acc 65.1%	Small dataset; limited to ML
22	Rachidi et al. (2023)	ML (SVM, NB, RF, LR) and DL (LSTM)	Instagram Moroccan dialect	Moroccan Arabic	LSTM Acc 83.64%; SVM Acc 75.04%	Scarcity of tools/datasets for dialect; modest results
23	Alrashidi et al. (2023)	Fine-tuned Arabic BERT, Multi-task Learning	Multi-aspect abusive tweets dataset	General Arabic	MTL + BERT > DL baselines; GitHub data shared	Imbalanced datasets; Arabic only
24	Elzayady et al. (2023)	CNN-LSTM, CNN-BiLSTM, CNN-GRU, AraBERT +Personality Features	Twitter hate speech dataset	General Arabic	AraBERT + personality features Acc 82.3%; CNN-LSTM 77%	Personality inference adds complexity; dataset size moderate
25	Khezzar et al. (2023)	LR, SVC, DT, CNN, AraBERT; web app (arHateDetector)	arHateDataset (merged public sets), Twitter	Standard + dialectal Arabic	AraBERT accuracy 93%; precision/recall/F1 reported	Aggregated datasets may introduce label/definition drift; external validation not detailed
26	Alsafari et al. (2020a)	Single and ensemble CNN/BiLSTM; AraBERT vs. non-contextual embeddings	Twitter; fine-grained two/three/six-class corpora	Mixed Arabic dialects	Ensemble F1: 91% (2-class), 84% (3-class), 80% (6-class); AraBERT > non-contextual; CNN > BiLSTM	Class granularity increases difficulty; error analysis shows issues with implicit/defensive language
27	Aljuhani et al. (2022)	BiLSTM with domain-specific embeddings; LR, SVM baselines	Tweets (seeded crawl, cleaned, labeled)	General Arabic (Twitter)	LR on char n-grams P/R/F1 = 92%; SVM ≈ 90%; BiLSTM competitive with domain embeddings	Seed-term collection bias; translation/generalization across topics not assessed
28	Amer Hamzah and Dhannoon (2023)	BiLSTM + Temporal Convolutional Network (TCN)	CASH: tweets on sexual harassment	Sexual-harassment domain	Accuracy 96.65%; F0.5 = 0.969; > XGBoost baseline	Task/domain specific; dialectal robustness not analyzed
29	Boulouard et al. (2022)	BERT EN, AraBERT, mBERT (AR/EN), LinearSVC, LSTM	YouTube comments (Gulf, Egyptian, Iraqi); Tweets	Mixed Arabic dialects; EN translations	BERT EN Acc 98%; AraBERT Acc 96%; mBERT-AR Acc 83%; LSTM Acc 82%	Translation pipeline may inflate EN results; sarcasm remains challenging
30	Aljarah et al. (2021)	SVM, NB, DT, RF; feature sets (TF-IDF, profile, emotion)	Twitter	General Arabic (varied topics)	RF best: Acc/G-mean 0.910; Recall 0.923; Precision 0.902 with all features	Small corpus after filtering; two-annotator protocol; neutrals excluded from training
31	Mouheb et al. (2019)	Naïve Bayes	Twitter + YouTube	General Arabic	Accuracy 0.959	Small dataset; limited feature diversity
32	Alakrot et al. (2021)	LR, SVM/LinearSVC, NB, DT, RF; POS + n-grams; feature selection	YouTube comments	Mixed dialects (YouTube)	LinearSVC highest accuracy (reasonable overall); gains from feature selection	Focus on offensive, not CB; dependence on preprocessing choices
33	Omar et al. (2021)	LinearSVC, NB variants, SVM, LR, DT, SGD, RF; multilabel pipeline	OSN posts across 11 classes; vulgar-speech set	General Arabic (Facebook/Twitter)	With Chi-square FS: Acc 97.92%; F1 97.92%; Precision 97.92%; Recall 97.93%; multilabel LinearSVC + TF-IDF Acc 82.29%, F1 92.48%	High feature counts; results sensitive to FS; generalizability outside OSN mix not shown
34	Shannaq et al. (2022)	Word-embedding fine-tuning + GA-optimized SVM/XGBoost	ArCybC (CB/Non-CB/Off/Non-Off)	Twitter; cyberbullying + offensive	SVM Acc 86.5% → 87.5%; XGB Acc 84.9% → 85.2% after optimization	Incremental gains; relies on a single public corpus
35	Kanan et al. (2021)	Unsupervised K-Means vs. EM (clustering)	(Facebook/Twitter)	General Arabic	Evaluated via training time, SSE (e.g., 7,796.363), and log-likelihood (e.g., 3,606.4669)	No precision/recall/F1; clustering quality hard to align with downstream moderation needs

#### 6.1.2 Sentiment analysis and lexicon-based methods

Sentiment analysis, often coupled with lexicon-based approaches, is commonly used to detect harmful content. AlHarbi et al. (2019) and Farid and El-Tazi (2020) used sentiment-based lexicons for Arabic texts, finding that pointwise mutual information (PMI) and lexicon enhancement can improve detection accuracy. Sentiment-based approaches are also utilized alongside NLP tools, such as tokenization and stemming, for feature extraction, enhancing the ability to detect cyberbullying based on emotional cues.

#### 6.1.3 Handling Arabic dialects and linguistic complexity

Dialectal Arabic presents a significant challenge, as standard ML models may not perform well on diverse dialects. Studies such as Alsubait and Alfageh (2021) and Al-Hassan and Al-Dossari (2022) indicate that datasets tailored to specific dialects (e.g., Egyptian, Levantine) enhance detection efficacy. Additionally, transformer-based models like AraBERT and multilingual BERT have emerged as effective tools for dealing with dialectal variations, as they can better capture semantic nuances across dialects (e.g., Khezzar et al., 2023; Alrashidi et al., 2023).

### 6.2 Research question 2

How has cyberbullying been detected in previous studies based on standards that represent its definition and characteristics?

The following themes were developed to answer the second research question.

#### 6.2.1 Development and use of cyberbullying datasets

Arabic cyberbullying detection relies heavily on curated datasets. Studies often use platform-specific datasets from Twitter, YouTube, and Facebook, with datasets labeled for harmful or offensive language (e.g., Bashir and Bouguessa, 2021; Khairy et al., 2023). These datasets include common cyberbullying characteristics like threats, insults, and hate speech. However, the issue of dataset imbalance (more non-cyberbullying content than cyberbullying) persists, affecting model performance. Techniques like oversampling and downsampling have been employed to address this imbalance, as seen in AlFarah et al. (2022). Table 3. Shows some examples of the existing datasets addressing cyberbullying in Arabic.

**Table 3:** Examples of the datasets addressing cyberbullying in Arabic.

Dataset (year)	Platform	Labels	Study
Instagram-Based Benchmark Dataset for Cyberbullying in Arabic (2022)	Instagram	Comments collected; multi-class sub-categories for bullying with sentiment variants used in evaluation (incl. Positive/negative/neutral)	Albayari and Abdallah (2022)
ArCybC / ArCyC—Arabic Cyberbullying Corpus (2022 article; 2023 data release)	Twitter (X)	Tweets; dual annotation tasks: CB vs. non-CB and Offensive vs. non-Offensive; 5 annotators	Shannag et al. (2022)
ArbCyD—Arabic Post Dataset for Cyberbullying Detection (2024)	Twitter (X)	Posts: bullying vs. non-bullying binary labels	Aljalaoud et al. (2025)

The ArCybC/ArCyC corpus represents one of the few openly accessible multi-dialect Twitter datasets that makes a clear distinction between cyberbullying and general offensive content. Its development is supported by detailed documentation of the annotation pipeline and guidelines, ensuring methodological transparency (Shannag et al., 2022). The ArbCyD dataset significantly expands the available volume by including annotated Twitter posts (Aljalaoud et al., 2025).

#### 6.2.2 Standards and evaluation metrics

Standards such as precision, recall, F1-score, and accuracy are commonly used to evaluate detection methods (e.g., Haidar et al., 2017; Alakrot et al., 2018). Although precision and recall are essential for accurate detection, the unique characteristics of the Arabic language and cyberbullying-specific terms often require additional metrics and customized standards. Studies such as El-Alami et al. (2022) and Amer Hamzah and Dhannoon (2023) advocate for using contextual features like sentiment polarity, emojis, and user history in cyberbullying detection. These standards help capture the nuanced characteristics of online abuse, especially within specific platforms or dialects.

Some evaluations adopt three-way labeling schemes that distinguish bullying/abusive content, non-bullying content, and neutral content. When overall accuracy is computed across all classes, the typically high prevalence of neutral instances can inflate the metric and obscure a system’s effectiveness on the bullying class, which is the primary target in safety-critical applications. For example, the Instagram-based Arabic cyberbullying benchmark provides a multi-class design with positive (bullying), negative (non-bullying), and neutral categories, together with inter-annotator agreement reporting and baseline models (Albayari and Abdallah, 2022). In such settings, macro-F1 and per-class F1 are preferable for comparing systems intended to detect bullying, whereas accuracy across all three classes can be misleading when neutral content dominates the distribution.

#### 6.2.3 Application of linguistic and psychological standards

Recent research has incorporated psychological theories to enhance cyberbullying detection by analyzing underlying personality traits in text (e.g., Elzayady et al., 2023). Such frameworks align detection methods with broader behavioral standards, moving toward a more human-centered approach in identifying abusive content. Other studies, such as Boulouard et al. (2022), address multilingual standards by analyzing Arabic text in translation and leveraging cross-linguistic BERT models, thus ensuring consistency in detecting cyberbullying characteristics across languages.

### 6.3 Research question 3

The third RQ was:

What future research directions in cyberbullying detection may be established based on the findings of the provided systematic review?

The following themes were developed to answer the third research question.

#### 6.3.1 Expansion of dialect-specific datasets and multilingual analysis

Future research could focus on developing larger, dialect-specific datasets to address the significant linguistic diversity in Arabic. Datasets for Moroccan, Syrian, and Gulf dialects remain limited and would improve detection accuracy for specific regions (e.g., Rachidi et al., 2023; Ali and Kurdy, 2022). Studies also suggest expanding multilingual capabilities to improve cross-linguistic performance, with transformer models like BERT and mBERT showing potential for multilingual hate speech analysis (e.g., Alrashidi et al., 2023; Shannaq et al., 2022).

For limited-resource settings, few strategies with large language models can be grounded in complementary lines of evidence. First, in-context learning has been shown to deliver strong few-shot performance without gradient updates; GPT-3’s original study established that scaling enables task-agnostic adaptation via a handful of exemplars embedded in the prompt, a result that has shaped subsequent methodology for low-data regimes (Brown et al., 2020). Second, prompt-based and prompt-free fine-tuning methods consistently improve over naïve fine-tuning when labeled data are scarce. Pattern-Exploiting Training and its generative extension reframe supervision as cloze-style patterns to amplify supervision from very small datasets, while LM-BFF automates prompt construction and demonstration selection to yield large gains across classification and regression tasks (Schick and Schütze, 2020). Complementing these, SetFit avoids handcrafted prompts altogether by contrastively fine-tuning sentence-transformer encoders on a handful of pairs and then training a lightweight classifier on the induced embeddings, matching or surpassing larger fully fine-tuned models under strict few-shot budgets (Tunstall et al., 2022). Moreover, parameter-efficient adaptation techniques such as LoRA reduce trainable parameters by orders of magnitude while preserving or improving downstream quality, which is particularly attractive when domain transfer must be achieved under tight compute and annotation constraints (Hu et al., 2022). To mitigate the scarcity of human-written instructions, Self-Instruct bootstraps synthetic instruction–input–output triplets from the model itself and shows substantial gains over the base model, offering a practical path when labeled data are limited (Wang et al., 2022). Evidence from multilingual and domain-specific studies indicates that these approaches translate beyond English benchmarks. Cross-lingual in-context learning studies report consistent benefits for genuinely low-resource languages and highlight alignment techniques that stabilize label semantics across languages, while evaluations in biomedical and clinical NLP show that instruction-tuned LLMs can perform competitively on few-shot entity recognition, QA, and relation extraction when carefully prompted (Cahyawijaya et al., 2024).

#### 6.3.2 Enhanced deep learning models and feature engineering

Future research could involve advancing feature engineering, particularly through contextual embeddings, attention mechanisms, and personality inference models. These methods could enhance the interpretability of cyberbullying detection systems and better capture contextual aspects of offensive language (e.g., Mohaouchane et al., 2019; Elzayady et al., 2023). Additionally, hybrid models combining CNN, RNN, and BERT-based architectures have shown promise for handling complex language features, and future studies could explore further model fusion or ensemble methods for improved accuracy (e.g., Mohaouchane et al., 2019; Althobaiti, 2022).

#### 6.3.3 Ethical considerations and real-time detection systems

Ethical standards and privacy concerns will play a growing role in future cyberbullying detection research. Privacy-preserving algorithms, especially those that anonymize or filter sensitive information, can support ethical AI use on social media platforms (e.g., Omar et al., 2021). Another area for future exploration is real-time cyberbullying detection systems that respond dynamically to harmful content as it is posted. While challenging, real-time models could be feasible with lightweight DL architectures tailored for social media monitoring (e.g., Amer Hamzah and Dhannoon, 2023; Kanan et al., 2021).

Ethical risks arise at each stage of dataset development and deployment for Arabic cyberbullying detection, beginning with data collection. The Instagram-based benchmark demonstrates the value of reporting annotation protocols and inter-annotator agreement alongside careful corpus descriptions; however, as with Twitter- and YouTube-based datasets, the presence of user mentions and cross-post threads can inadvertently expose targets and perpetrators if not aggressively sanitized (Albayari and Abdallah, 2022; Haidar et al., 2019; Alakrot et al., 2018; Alduailaj et al., 2023; Al-Ajlan and Ykhlef, 2018; Alrougi et al., 2024). Representativeness is a second, persistent ethical and scientific concern. Arabic social media is heterogeneous across dialects, platforms, and communities; yet several widely used datasets skew toward particular dialect clusters or platform norms, such as Egyptian or Gulf Twitter, pan-Arab YouTube comments, or Instagram captions from specific demographic groups (Haidar et al., 2019). Studies that publish clear guidelines, show label distributions, and report inter-annotator agreement support more accountable modeling than those that provide only aggregate scores (Albayari and Abdallah, 2022). Curators should also protect annotator wellbeing through workload limits, content warnings, and access to support, and they should state these safeguards in their documentation. The evaluation protocol has ethical implications because metric choice shapes decision thresholds used in practice. Practical architectures therefore favor lightweight normalizers and dialect-aware tokenization before model inference, with privacy-preserving logging that stores only hashed text fingerprints or short-lived embeddings for auditing (Alakrot et al., 2018). The more explicit dataset papers are about these elements, the less likely it is that downstream models will inadvertently encode representational harms or privacy leakage.

#### 6.3.4 Integration of psychological and social dimensions

Integrating psychological and social analysis within detection algorithms is emerging as an essential direction. Personality-based approaches could be particularly useful, helping identify users more likely to engage in or be affected by cyberbullying (e.g., Elzayady et al., 2023).

Additionally, cross-disciplinary research involving psychology, sociology, and computational linguistics could establish standards for understanding the social dynamics underlying cyberbullying, offering insights beyond linguistic patterns (e.g., Omar et al., 2021). Table 4 shows the summary of the themes related to each research question.

**Table 4:** Summary of the themes related to each research question.

Research Question	Theme	Description	Sources
RQ1: Current trends in cyberbullying detection for Arabic language and dialects	ML and DL Approaches	ML models (e.g., SVM, Naïve Bayes) and DL models (e.g., CNN, BERT) are common for cyberbullying detection, with ensemble methods improving accuracy.	Haidar et al. (2017); Alakrot et al. (2018); Alrashidi et al. (2023)
	Sentiment Analysis and Lexicon-Based Methods	Sentiment analysis and lexicon-based approaches capture emotional tones and harmful language, essential for handling Arabic’s diverse dialects.	AlHarbi et al. (2019); Farid and El-Tazi (2020)
	Handling Arabic Dialects and Complexity	Specialized datasets and models (e.g., AraBERT, multilingual BERT) address dialectal variability, enhancing model accuracy for Arabic.	Mubarak and Darwish (2019); AbdelHamid et al. (2022); Khezzar et al. (2023)
RQ2: Standards used for detecting cyberbullying based on its characteristics	Development of Cyberbullying Datasets	Creation of Arabic-specific datasets that include dialectical variations and cyberbullying characteristics, though issues like imbalanced datasets (few cyberbullying instances) impact model performance.	Bashir and Bouguessa (2021); Khairy et al. (2023); AbdelHamid et al. (2022)
	Evaluation Standards and Metrics	Precision, recall, F1-score, and accuracy are commonly used metrics, supplemented by specialized metrics tailored to Arabic-language characteristics to ensure reliable detection performance.	Haidar et al. (2017); Alakrot et al. (2021); Boulouard et al. (2022)
	Linguistic and Psychological Standards	Integration of linguistic and psychological insights, such as personality inference, allows a deeper understanding of user behavior, helping to identify cyberbullying based on more human-centered behavioral traits.	Elzayady et al. (2023); Omar et al. (2021); Shannaq et al. (2022)
	Contextual and Cultural Considerations	Incorporation of cultural sensitivity, including the use of dialect-specific language features, emojis, and contextual sentiment, provides a more nuanced and culturally accurate detection of offensive language.	AlHarbi et al. (2019); Farid and El-Tazi (2020); Khezzar et al. (2023)
RQ3: Future research directions for Arabic cyberbullying detection	Dialect-Specific Datasets and Multilingual Models	Expansion of dialect-specific datasets and multilingual models to enhance detection across Arabic dialects and improve cross-linguistic applicability.	Ali and Kurdy (2022); Rachidi et al. (2023); Shannaq et al. (2022)
	Advanced Feature Engineering and Hybrid Models	Development of hybrid models (e.g., CNN-LSTM-BERT) and advanced feature engineering, such as attention mechanisms and personality-based features, for richer context and improved detection accuracy.	Mouheb et al. (2019); Elzayady et al. (2023); Boulouard et al. (2022)
	Real-Time Detection and Privacy Considerations	Focus on real-time cyberbullying detection models for immediate response, with privacy-preserving techniques to ensure user data protection and ethical AI application.	Amer Hamzah and Dhannoon (2023); Omar et al. (2021); Kanan et al. (2021)
	Cross-Disciplinary Research	Integration of psychological, sociological, and linguistic insights for a more comprehensive understanding of the social and behavioral dynamics underlying Arabic cyberbullying.	Farid and El-Tazi (2020); Omar et al. (2021); Elzayady et al. (2023)

The results of the research emphasize the necessity of culturally sensitive detection models, sophisticated methodologies, and tailored approaches to effectively capture the distinctive characteristics of the Arabic offensive language. Arabic is an extremely diverse language, with significant variations in dialects across regions (e.g., Egyptian, Gulf, Levantine), each with its own vocabulary, syntax, and expressions. The detection of objectionable language is further complicated by this diversity, as models that have been trained in Modern Standard Arabic frequently encounter difficulties with dialectal content. These results suggest that the model’s ability to identify nuanced or implicit forms of offensive language, such as sarcasm or mockery, is improved by the inclusion of sentiment and lexicon-based features that are specifically designed for Arabic dialects and slang. Many categories of offensive language, including religious hate speech, ethnic hate, and political offence, have been classified by researchers. These types of language are particularly sensitive in Arabic-speaking societies. These categories are indicative of regional and cultural priorities, emphasizing the social and religious values that influence online discourse in Arabic contexts. The importance of accounting for these categories is underscored by research, as they pertain to highly sensitive subjects that may vary in severity and context in comparison to other languages. The results indicate that culturally aware models that identify these particular forms of objectionable language can improve the accuracy and relevance of the models.

Although numerous studies have examined cyberbullying detection methods broadly or across various languages, there is a paucity of focused analyses on Arabic-language detection, given the unique challenges presented by Arabic’s morphological intricacies and dialectal diversity (Mubarak and Darwish, 2019; AbdelHamid et al., 2022). The majority of the earlier studies predominantly analyze general patterns in cyberbullying detection, concentrating on English-language research (Alakrot et al., 2018; Bashir and Bouguessa, 2021). Although current studies recognize dataset imbalances and biases in social media-derived training data, they frequently neglect to consider privacy concerns and the ethical ramifications of automated cyberbullying detection among Arabic-speaking groups (Omar et al., 2021; Amer Hamzah and Dhannoon, 2023). This study addresses real-time detection concerns, the balance between moderation and free speech, and the necessity for privacy-preserving machine learning algorithms in social media monitoring (Kanan et al., 2021). This paper distinctly focuses on the thorough assessment of ML and DL models in detecting cyberbullying in Arabic. The prior systematic literature review by Castaño-Pulgarín et al. (2021), addressed cyberbullying detection on studies that provided exploratory data about the Internet and social media as a space for online hate speech, types of cyberhate, terrorism as an online hate trigger, online hate expressions and the most common methods to assess online hate speech. Balakrisnan and Kaity (2023) also did an SLR focusing on three main areas regarding cyberbullying detection through machine learning: the algorithms employed, the features used for detection, and the performance measures of these methods. The prior studies and reviews neglect Arabic-specific issues such as root-based word creation, tokenization complexities, and script intricacies.

The results of this study underscore the necessity of creating extensive, dialect-specific datasets and enhancing NLP models to address syntactic and lexical discrepancies among Arabic dialects. Deep learning architectures such as CNNs and BiLSTMs generally surpass classical baselines once training sets exceed the low-thousands and when preprocessed to handle orthographic variation, elongation, and code-mixing. Transformer models fine-tuned on Arabic corpora—especially variants trained with substantial dialectal coverage—consistently lead when the label definitions align with the pretraining distribution and when macro-averaged F1 rather than accuracy guides optimization. A recurring empirical pattern is precision outpacing recall, reflecting systems that confidently flag explicit bullying but struggle with implicit attacks, sarcasm, and context-dependent harassment. Performance differences are driven first by data composition. Dialectal diversity, platform genre, and class design are the most decisive factors. Models trained on tweets in Egyptian or Gulf dialects tend to degrade on Levantine, Maghrebi, or code-mixed content because lexical cues and morphological patterns shift, and subword tokenizers learned on Modern Standard Arabic under-segment dialectal forms. Domain shift between platforms—short, slang-heavy tweets versus longer Instagram captions or YouTube comments—likewise reduces transfer, as does the prevalence of emojis, creative spellings, and Arabizi. Class definitions also vary: some corpora equate cyberbullying with general abuse or toxicity, whereas others require intent, repetition, or power imbalance. The broader the “bullying” label, the higher the apparent scores, but the weaker the comparability across studies. Evaluation choices amplify these effects. Where annotation guidelines were explicit and inter-annotator agreement documented, models learned more stable decision boundaries; where guidelines were minimal or borrowed from sentiment analysis, models overfit to superficial polarity and miss community-specific bullying norms. Pretraining and representation learning explain the remaining variance. Yet, when fine-tuning data are severely imbalanced, even strong encoders prioritize surface toxicity over nuanced bullying constructs. In contrast, classical models augmented with curated lexicons and character-level features sometimes outperform deep baselines on noisy, low-resource dialects because they are less sensitive to tokenization errors and require fewer examples to generalize.

The most promising methodological direction is dialect- and domain-robust modeling anchored in standardized evaluation. Progress depends on a benchmark suite that harmonizes label schemas for cyberbullying versus general abuse, publishes class priors, and mandates macro-F1 and per-class F1 with clear treatment of the neutral class. Cross-dataset testing should be routine, with models trained on one corpus evaluated zero-shot on another to measure real-world robustness. Data and supervision strategies also offer leverage. Active learning and disagreement-focused annotation can densify minority bullying phenomena such as threats, doxxing, or body-shaming. Weak supervision that combines lexicon rules, community guidelines, and pattern matchers can cheaply label large pools for pretraining, followed by human verification on hard examples. Span-level rationales and multi-label tags for bullying types improve transparency and enable error analysis beyond single-label outcomes, while adversarial training with paraphrases and sarcasm transformations hardens models against implicit aggression. Context modeling is a further frontier. Many failures stem from sentence-level isolation. Incorporating conversation threads, author–target history, and lightweight social signals can disambiguate teasing from harassment and detect repetition, a hallmark of bullying. Graph-based representations of interactions, when coupled with privacy-preserving design and strict ethical safeguards, can capture power asymmetries and coordinated attacks without storing sensitive personal attributes.

Finally, instruction-tuned large language models adapted to Arabic show potential as few-shot labelers, error analyzers, and data generators, but their deployment must be paired with rigorous calibration, bias auditing across dialects and demographics, and conservative thresholding in safety-critical pipelines. Taken together, the evidence suggests that the field is moving from accuracy on single, homogeneous datasets toward robust, dialect-inclusive systems evaluated under standardized, recall-sensitive protocols, with the integration of context and improved supervision likely to yield the next substantive gains.

## 7 Limitations and suggestions for future studies

A key limitation of this review is the absence of a formal quality appraisal or risk-of-bias assessment of the included studies. Established tools such as AMSTAR, AMSTAR-2, or ROBIS are often used in systematic reviews to evaluate the methodological rigor of primary studies and to distinguish between stronger and weaker evidence. The present review treats all included studies as methodologically equivalent, regardless of variations in their design, sampling strategies, or analytical robustness.

The majority of the studies reviewed are based on restricted or specific datasets, which may not adequately represent the complete range of Arabic dialectal diversity or the diverse forms of cyberbullying that are present on different platforms. However, the absence of standardized datasets for the detection of Arabic cyberbullying also presents obstacles to the attainment of generalizable results. Despite the potential of dialect-specific models, the complexity and extensive variations among Arabic dialects pose a significant obstacle. The results may not be broadly applicable because current models may not perform consistently across all dialects. The detection of real-time cyberbullying is still in its infancy, particularly in the context of Arabic texts. Although some studies incorporate psychological insights, there is a void in the comprehensive integration of insights from sociology, linguistics, and psychology to develop a holistic understanding of cyberbullying behaviors specific to Arabic-speaking regions. Another limitation of this review is the exclusion of conference proceedings, despite their prominence as venues for innovation in natural language processing. Nonetheless, this exclusion may have led to the omission of some cutting-edge contributions. Future reviews should consider incorporating both journal articles and high-quality conference proceedings to present a more comprehensive view of the research landscape.

Future research may investigate sophisticated deep learning architectures and hybrid models that amalgamate various methodologies to enhance detection, to improve contextual comprehension and classification precision. Another vital avenue for future study is the enhancement of sentiment-based and context-aware models for detecting cyberbullying. The problem of dataset imbalance persists, as cases of cyberbullying are markedly underrepresented relative to non-offensive content.

## 8 Conclusion

This study offers a thorough examination of the most recent academic research, methodologies, and challenges in the detection of cyberbullying in Arabic texts. This review emphasizes the substantial advancements that have been achieved in this field by evaluating the efficacy of ML and DL models, sentiment analysis, lexicon-based methods, and dialectal considerations. The significance of specialized datasets for Arabic dialects, the efficacy of composite models and ensemble learning, and the value of sentiment-based and contextual analysis are underscored by the key findings.

## Data Availability

The original contributions presented in the study are included in the article/supplementary material, further inquiries can be directed to the corresponding author/s.
